# Associations between mandibular torus and types of temporomandibular disorders, and the clinical usefulness of temporary splint for checking bruxism

**DOI:** 10.1186/s12903-021-01550-y

**Published:** 2021-04-09

**Authors:** Hee-Min Lee, Dong-Woo Kang, Pil-Young Yun, Il-hyung Kim, Young-Kyun Kim

**Affiliations:** 1 Department of Oral and Maxillofacial Surgery, Section of Dentistry, Seoul National University Bundang Hospital, 82 Gumi-ro 173beon-gil, Bundang-gu, Seongnam, 13620 Korea; 2 Office of Human Resources Development, Armed Forces Capital Hospital, Armed Forces Medical Command, Seongnam, Korea; 3 School of Dentistry and Dental Research Institute, Seoul National University, Seoul, Korea

**Keywords:** Mandibular torus, Temporomandibular disorders, Oral parafunctional habits, Bruxism, Clenching, Splint, Temporary splint for checking bruxism

## Abstract

**Background:**

Occlusal stress from oral parafunctional habits is one of the causes of temporomandibular disorders (TMD) and mandibular torus (MT). Although some studies have investigated the correlation between TMD and MT, understanding of the relationships between types of TMD and MT is insufficient. Therefore, we conducted this study to investigate the associations between presence of MT and TMD types.

**Methods:**

This study included 77 patients diagnosed with TMD who first visited our clinic for TMD between March 2019 and July 2020. Among them, 30 (38.9%) had MT, and 54 (70.1%) had oral parafunction. Parafunctional activity during sleep was confirmed using a temporary splint for checking bruxism (TSCB).

**Results:**

The relationship between prevalence of MT and oral parafunction in TMD patients was not statistically significant (*P* = 0.131), but the odds ratio was relatively high at 2.267. An analysis of TMD type revealed that Type I, which is classified as myalgia of the masticatory muscles, and MT had a significant association (*P* = 0.011). We fabricated a TSCB for 27 patients to wear during sleep and confirmed that 23 (85.2%) had nocturnal bruxism. The TSCB results and presence of MT showed a significant relationship (*P* = 0.047).

**Conclusion:**

Through the results of this study, clinicians may consider the hyperactivity of masticatory muscles in the presence of MT when treating TMD patients. In addition, TSCB has a great diagnostic value as it can be easily manufactured and be useful for discovering pre-existing oral parafunctions that patients are not aware of.

## Background

Temporomandibular disorders (TMD) are a group of disorders of the masticatory muscles of the temporomandibular region, joints, and related anatomical structures. TMD results from macrotrauma and microtrauma; the latter occurs when a load is repeatedly applied to a joint’s structure over a long period of time, resulting in destruction due to hypoxia from insufficient blood flow [1]. Masticatory muscle pain caused by excessive use also is considered a cause of TMD [2]. Swelling occurs as a result of increased intramuscular pressure and continuous activation of several motor units to produce a lowered threshold for excessive load. When these muscles are damaged, symptoms of inflammation such as pain, swelling, and tenderness occur in the temporomandibular region [3]. Therefore, among various causes, microtrauma continuously applied to the region due to excessive masticatory function and occlusal stress due to oral parafunction is a major risk factor for TMD [4, 5].

Mandibular torus (MT) is a benign bone growth with clear border and slow growing rate. It mostly appears on lingual side of bilateral mandible premolar. Although the cause of MT is not understood, occlusal stress and excessive masticatory function have been suggested as the major factors [6–8]. Therefore, it is convincing that oral parafunctions such as clenching or teeth grinding are closely related to the occurrence of MT as well as TMD. Some studies have reported the relationship among TMD, oral parafunctions, and MT [6, 9], while some studies have reported on the relationship between oral parafunctions and MT [7, 9, 10].

Nevertheless, studies have not focused on the relationship between subtype of TMD and of MT. Therefore, in this study, we classified TMD patients by subtype and analyzed the concordant presence of MT. Through this study, we would like to highlight the MT’s diagnostic value hidden in, which is often overlooked in clinical practice. Also, we would like to introduce a simple and useful device called Temporary Splint for Checking Bruxism (TSCB) that we use to diagnose TMD patients.

## Methods

### Study subjects

The subjects were patients diagnosed with TMD who first visited the Oral and Maxillofacial Surgery Department of Seoul National University Bundang Hospital between March 2019 and July 2020. The criteria for excluding research subjects were as follows: (1) history of maxillofacial tumor, (2) history of facial trauma, (3) absence of one or more molars that had not been restored, and (4) history of botulinum toxin injection within the last six months. The study was approved by the Seoul National University Bundang Hospital Institutional Review Board (IRB) (B-2010-642-104) and was performed in accordance with the Helsinki guidelines. Informed consent was obtained from all subjects.

### Research category

#### Temporomandibular disorders

All the subjects were diagnosed with TMD by one calibrated and criterion standardized oral and maxillofacial surgeon who performed the same clinical exam for each. The initial visit consisted of a questionnaire on their pain level and duration as well as clinical examination on TMD-related symptoms using Diagnostic Criteria for Temporomandibular Disorders (DC/TMD). Then, orthopantomography and temporomandibular panoramic radiography were taken for radiological examination and cone beam computed tomography (CBCT) and bone scan or single photon emission computed tomography (SPECT) were used as needed. After every inspection, patients were diagnosed as one or more of TMD type I to IV, a detailed diagnostic criteria based on the TMD classification system of the Japanese Society for Temporomandibular Joint (JSTMJ, 2013). According to the system, TMD is classified into myalgia of the masticatory muscle (Type I), arthralgia (Type II), disc derangement (Type III), and osteoarthrosis or osteoarthritis (Type IV) (Table 1). Each type in this taxonomy corresponds to a classification of DC/TMD, and Type I is masticatory muscle disorder, and Type II, III, IV are joint pain, joint disorders, and joint diseases respectively in temporomandibular joint disorders.

**Table 1:** Classification of temporomandibular joint disorders (2013, JSTMJ^*^)

Type	Contents
I	Myalgia of a masticatory muscle
II	Arthralgia of the temporomandibular joint
III	Temporomandibular joint disc derangement
	a. with reduction
	b. without reduction
IV	Osteoarthrosis/osteoarthritis of the temporomandibular joint

*Japanese Society for Temporomandibular Joint

#### Mandibular torus

Five dentists who had completed the training for this investigation performed oral examination during the first visit to determine the presence of a MT. Patients were classified to have MT only when bilateral nodular mass above the mylohyoid ridge one the lingual side of the mandible in the canine to premolar area was clearly distinguishable by naked eye.

#### Oral parafunctional habits

Patients were considered to have oral parafunctions if any one of the following condition was present: (1) Clenching and/or bruxing, day or night, either self-reported or reported by their sleeping partners, (2) Patients with positive results of Temporary Splint for Checking Bruxism (TSCB) with relevant symptoms and signs through questionnaire and clinical examination at the first visit.

#### Temporary splint for checking bruxism

No matter how much patients were aware of their own damaging oral habits, TSBC was fabricated and delivered to be worn during night time if they reported to have, pain or tenderness during palpation in the regions of masticatory muscle when they open their mouth immediately after waking up. In addition, if intraoral exam showed scalloped tongue, linea alba on the buccal mucosa, and multiple occlusal attrition, abfractions, and hypersensitivity teeth that cannot be explained as others, TSCB was prescribed assuming that the patient had oral parafunctions.

**Fig. 1:**
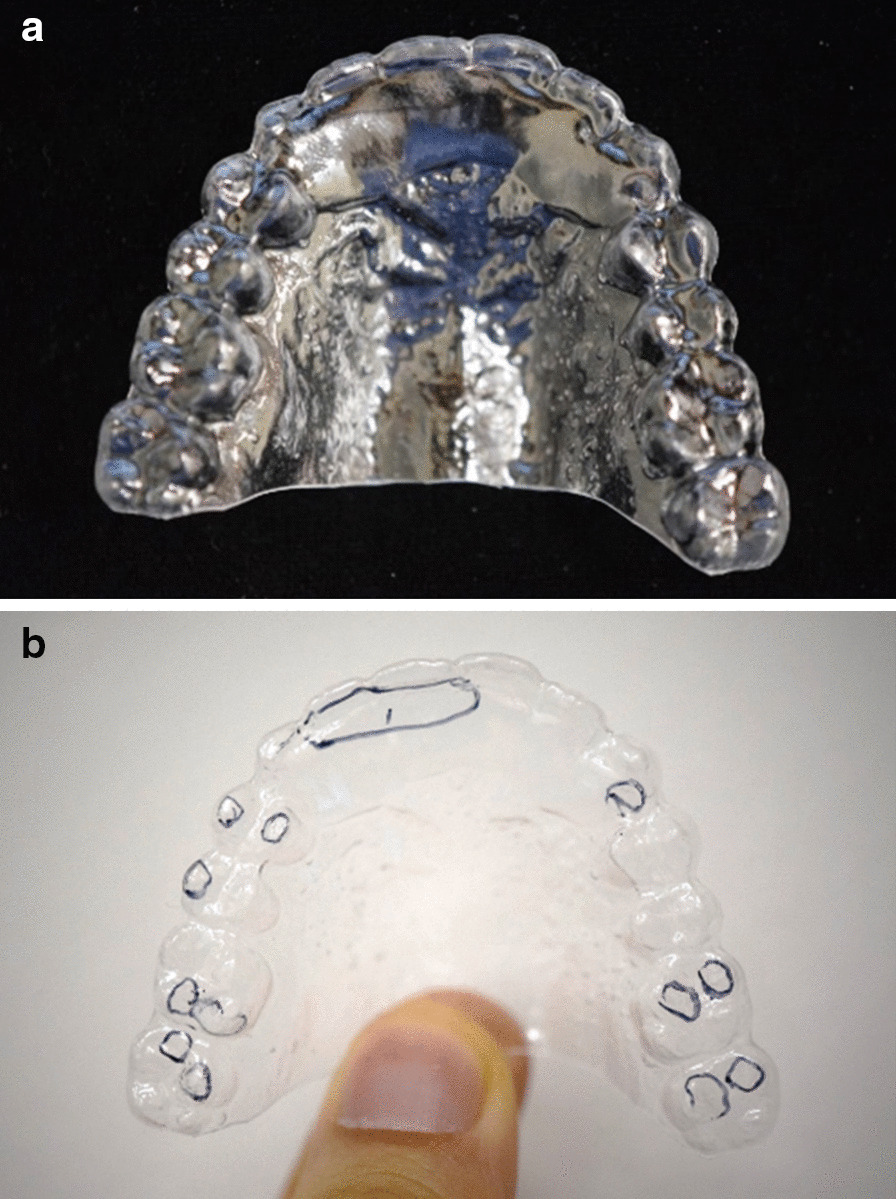
Temporary Splint for Checking Bruxism (TSCB) used to detect nocturnal oral parafunctions that patients are unaware of **a** The TSCB is a hard Omnivac splint with a 0.6-mm thickness. **b** After using the device during sleep for a week, several marks are identified on the surface, indicating that the patient is a bruxer at night

TSCB is a transparent and hard Omnivac splint with a thickness of 0.6 mm. Alginate impressions were taken at the first visit for the patients in need, and immediately fabricated and given to patients. After wearing it while sleeping for one week, the number of scratched or perforated points observed on the surface of the device at the next visit was applied as the result of TSCB (Fig. 1). Cases with more than one TSCB result were regarded to have oral parafunctions.

### Statistical analysis

For statistical analysis, IBM SPSS version 25.0 (SPSS Inc., Chicago, IL, USA) was used, and the statistical significance level was set to 95%. Statistical analysis was performed on sex, age, duration of TMD-related symptoms, TMD type, oral parafunction, and TSBC results for the presence or absence of MT. Logistic regression analysis was used for analysis of sex, age, and TMD type, and a chi-square test was carried out to analyze oral parafunction. For analysis of duration of TMD-related symptoms and TSBC results, a linear-by-linear association was used.

## Results

The study comprised 77 patients, all Koreans: 27 males (35.1%) and 50 females (64.9%) with an average age of 41.7 (13–84) years. Of the total patients, 30 (38.9%) had MT, and 54 (70.1%) demonstrated oral parafunction. The average age of patients with MT was 37.7 (13–67) years. In addition, 11 of 27 males (40.7%) and 19 of 50 females (38.0%) had MT. Oral parafunction was confirmed in 20 of 27 males (74.1%) and in 38 of 50 females (76.0%). Age and sex were not significantly associated with MT. In addition, there was no statistical significance between MT and oral parafunction, but the odds ratio was relatively high at 2.267 (Table 2).

**Table 2:** Demographics, mandibular torus and oral parafunctions in patients with TMD

	Male	Female	Total	*P*-value
Age	43.8	40.7	41.76	0.108
Gender	27 (35.1%)	50 (64.9%)	77 (100%)	0.817
Parafunctions	17/27 (34.5%)	37/50 (76.0%)	54/77 (70.1%)	0.131
Mandibular torus*	11/27 (40.7%)	19/50 (38.0%)	30/77 (38.9%)	–

*Age, gender, and parafunctions were not statistically significant with mandibular torus, respectively (*P* > 0.05)

In the subjects, there were 10 distinct combinations of TMD types, with Types I and III being the most common (Table 3). When classifying the participants by TMD type, Type I TMD was the most common in 51 (49.0%) patients, followed by Type III in 44 (38.6%), Type IV in 33 (39.4%), and Type II in 8 (37.5%) participants. Statistical analysis confirmed that MT was significantly associated with TMD Type I (*P* = 0.011) (Table 4).

**Table 3:** Combinations by TMD types in the subject group

	I, III	I	I, III, IV	IV	III	I, IV	III, IV	I, II	II, III	I, II, III	Total
No. of patients	17	11	10	10	8	7	6	5	2	1	77

**Table 4:** Presence of mandibular torus according to each type of TMD

TMD type	Count	Presence of torus	*P*-value
I^*^	51	25 (49.0%)	0.011
II	8	3 (37.5%)	0.992
III	44	17 (38.6%)	0.856
IV	33	13 (39.4%)	0.428

^*^Statistically significant difference with mandibular torus (*P* < 0.05)

Interviews and clinical examinations of 77 TMD patients confirmed that 31 patients had parafunction. Of the remaining 46 patients, 27 were suspected to have oral parafunction and received a TSCB. After one week, 23 patients (85.2%) showed positive results on TSCB, and the score was 3.07 ± 2.67 out of 0 to 10 range. Overall, 54 patients were found to have oral parafunctional habits. It was confirmed that the increase of the TSCB result was statistically significant with the occurrence of MT (*P* = 0.047) (Table 5).

**Table 5:** Presence of mandibular torus according to the results of temporary splint for checking bruxism (TSCB) in patients with TMD

TSCB results^*^	0	1	2	3	4	5	6	7	8	10
No. of patients	4	7	3	2	4	2	2	1	1	1
No. of patients with torus	1 (25%)	2 (28.5%)	0 (0%)	0 (0%)	2 (50%)	2 (100%)	2 (100%)	1 (100%)	1 (100%)	0 (0%)

*Statistically significant association with mandibular torus (*P* < 0.05)

The duration of TMD-related symptoms investigated from the subjects was classified into less than 6 months, 6 months to 1 year, 1 to 2 years, 2 to 3 years, and 3 years or longer. Among them, 3 years or more was the most common, followed by less than 6 months, 1 to 2 years, 6 months to 1 year, and 2 to 3 years. No statistically significant value was observed for the correlation between the presence of MT and the duration of TMD-related symptoms (*P* = 0.940) (Table 6).

**Table 6:** Duration of TMD-related symptoms and presence of torus mandibularis in patient with TMD

Mandibular torus	Duration of symptoms^*^					
	< 6 months	6 months to 1 year	1 to 2 years	2 to 3 years	> 3 years	Sum
Present	6	4	5	4	11	30
None	11	4	9	3	20	47
Sum	17	8	14	7	31	77

*No statistically significant difference with mandibular torus (*P* < 0.05)

## Discussion

Several studies have reported a prevalence of bony exostosis in the oral cavity, including mandibular and palatal torus, and buccal exostosis, between 12 and 33% [11–13]. In particular, there is a higher incidence of MT among East Asians, Eskimoans, Mongolians, and Japanese; white people have a higher incidence than black people, demonstrating a large racial difference [14–17]. The most widely accepted theory of MT development is multi-factorial, including genetic and environmental factors. Eggen et al. reported MT to be affected by about 30% genetic factors and 70% environmental factors [18]. Other researchers have implicated factors such as trauma, drugs, infections, occlusal stress, and nutrition in occurrence of MT [19–21]. Among these, occlusal stress and excessive bite force have been presented as the main environmental factors involved in MT [6, 18].

Clifford et al. reported a close correlation between MT and parafunctional habits, and Sirirungrojying et al. suggested that MT could be used as a marker of TMD [6, 22]. In this study, the prevalence of MT (38.9%) in patients with TMD was higher than that (1.1 to 9.4%) in the general population [10, 15, 23, 24]. In addition, the predicted prevalence of sleep bruxism in general adults was 5.5 to 15.3% [25, 26], while the proportion of TSCB-proven sleep bruxism in TMD patients in this study was 29.9%. That is to say, TMD patients in this study showed higher prevalence of MT and nocturnal bruxism compared to the general population. This can be interpreted as a result of excessive and sustained occlusal force seen in TMD patients. We found no significant relationship between oral parafunctional habits and presence of MT in TMD patients, but the odds ratio had a high value of 2.267, which indicates a similar trend to the results of previous studies [6, 9, 22].

This study is the first to classify TMD patients by subtype and to investigate their relationship with MT. The taxonomic classification for TMD of JSTMJ used in this study, although it is unfamiliar in the Western world, is thought to be meaningful in that it helps TMD be understood by dividing it into four major categories. We confirmed that TMD Type I, which involves myalgia of the masticatory muscles, had a significant association with MT. Based on Wolff's Law, which holds that the bone undergoes remodeling as load increases and becomes stronger over time [27], this result can be explained. Singh et al. also argued that MT is developed by overworking of TMJ, leading to prolonged overload and activates osteoblasts to deposit bone, which seems to be a theory applicable to our results [28]. No meaningful association was found between duration of TMD symptoms and presence of MT in this study. As previously mentioned, MT occurs due to a persistent increased occlusal load, so it was expected that MT would be more frequent as TMD symptom retention period increased; however, our findings did not support this. Therefore, it is better to say that TMD is a complex disorder that should be understood by its subtype.

In this study, retention of MT according to sex in TMD patients was not significant. However, previous studies have shown that MT is diagnosed more often in women [12, 15, 17, 23, 24, 29]. This is thought to be due to the involvement of other factors contributing to the retention of MT since this study was conducted in patients diagnosed with TMD.

It has been negatively recognized whether electromyography (EMG) can be usefully used in diagnosing TMD [30, 31]. However, recent literatures have suggested that EMG values ​​of masticatory muscle have reliable diagnostic sensitivity in pain-related TMD patients [32, 33]. Although EMG was not used in this study, it would be better if EMG be used in the course of TMD treatment in the future by referring to these studies.

TSCB can both reveal and quantify the degree of unconscious oral parafunctions that occur at night. Since we were able to detect previously unnoticed oral parafunctions during sleep in 23 people (85.1%) by prescribing a TSCB to 27 patients, we suggest that this device be used as a simple but useful diagnostic tool. Our results indicate that the prevalence of MT significantly increases as TSCB results increase. Accordingly, the more severe is the abnormal oral function, the more common is development of MT. In addition, we found that patients who had used a TSCB had a very high rate of consent to continued stabilization splint treatment despite the high cost. Therefore, TSCB is a convenient and useful tool that can be used to diagnose and treat TMD.

MT and oral parafunctional habits were interrelated in TMD patients and were prominent in TMD Type I presenting masticatory muscle problems and these results can be useful when treating TMD patients. However, patients with MT are often unaware of its existence because of lack of symptoms or discomfort. Clinically, MT has not been considered important except in special situations, such as before the manufacture of dentures. Clinicians need to suspect that TMD patients with MT have parafunction and excessive activity in the masticatory muscle. This study is expected to increase understanding of MT and promote related research in the future.

## Conclusion

We confirmed a close relationship between TMD Type I and presence of MT. Moreover, we suggest that TSCB should be considered as a useful diagnostic device that can be easily manufactured and applied to provide more comprehensive diagnosis to TMD patients.

## Data Availability

The datasets used and/or analyzed in this study are available from the corresponding author upon reasonable request.
